# A BIO-EEG Hyperscanning Study of Moral Dyadic Negotiation

**DOI:** 10.3390/brainsci15091015

**Published:** 2025-09-19

**Authors:** Angelica Daffinà, Laura Angioletti, Michela Balconi

**Affiliations:** 1 International Research Center for Cognitive Applied Neuroscience (IrcCAN), Università Cattolica del Sacro Cuore, 20123 Milan, Italy; 2 Research Unit in Affective and Social Neuroscience, Department of Psychology, Università Cattolica del Sacro Cuore, 20123 Milan, Italy

**Keywords:** hyperscanning, moral negotiation, EEG, HRV, emotional-cognitive dynamics

## Abstract

Background/Objectives: Recent social neuroscience research has increasingly shifted from individual moral decision-making to the study of how people negotiate moral dilemmas in interpersonal contexts. This multimethod hyperscanning study investigated whether initial differences in moral decision-making orientation within a dyad influence neural and autonomic synchronization during a joint moral negotiation. Methods: Fourteen dyads were classified as homologous or heterologous based on the similarity or dissimilarity of their individual decision-making orientations. Each dyad was asked to negotiate and reach a shared decision on a moral dilemma involving a realistic health emergency scenario. Electroencephalography (EEG) and autonomic signals were recorded simultaneously. Dissimilarity indices were computed to assess inter-brain and autonomic synchronization. Results: EEG analyses revealed a significant effect only in the delta frequency band: all dyads, regardless of orientation, showed greater dissimilarity in the left frontal region compared to the left temporo-central and right parieto-occipital regions. In addition, autonomic data indicated greater heart rate variability (HRV) dissimilarity in homologous dyads than in heterologous ones. However, these results did not confirm our initial hypotheses, indicating the opposite pattern. Conclusions: Left frontal delta dissimilarity emerged as an exploratory candidate marker of moral negotiation across dyads. Greater HRV dissimilarity in homologous dyads suggests that, in these dyads, successful negotiation may be supported by complementary rather than synchronized autonomic responses. This multimethod hyperscanning approach highlights the complex and partially dissociable contributions of neural and autonomic processes to the regulation of shared moral decision-making.

## 1. Introduction

Traditionally, moral dilemmas are defined as situations involving choices between mutually exclusive alternatives, with each option carrying potential negative consequences [1]. Conventionally, the focus of research within this domain has been oriented towards the actions of a single decision-maker faced with a moral dilemma. However, recent developments in the field of neuroscience have prompted a shift in focus from individual moral decision-making to the study of moral dilemmas within the context of negotiation and cooperation between individuals [2,3]. This change represents a transition from the traditional individualistic and intrapsychic approach to moral dilemmas, towards a more socially oriented perspective. Nevertheless, there is a lack of research focusing on dyadic moral dilemmas, conceptualized as moral scenarios characterized by structural constraints requiring two individuals to collectively decide how to allocate limited resources or whom to save among equally deserving individuals [4,5]. Such scenarios are widely used in experimental settings and require decisions that are coherent not only on an intrapsychic level, but also through a dialogical process in which the two individuals are required to develop a shared interpretive and justifying model [6].

Consequently, dyadic moral judgment is increasingly understood as a phenomenon arising from the joint interaction and negotiation of the decision between the individuals involved in the process. In this context, negotiation, conceptualized as a complex process that incorporates the emotional and physiological elements of interaction, rather than merely representing a relational exchange, becomes crucial. Indeed, it involves the interplay of decision-making, collaborative problem-solving, and joint action to construct a shared meaning of the process [7,8,9].

While the most prominent neuroscientific studies on moral decision-making adopt a neurological localization approach, highlighting the central role of specific areas of the prefrontal cortex (PFC) [10,11,12], research that explores the electrophysiological (EEG) foundations of moral cognition has received less attention. The extant literature has primarily utilized event-related potential (ERP) analysis to investigate the temporal dynamics of moral cognition. For instance, some studies that have employed the Trolley or the Footbridge dilemma [13] have examined how factors such as legal responsibility [14] and dispositional empathy [15] modulate moral decision-making. In a relatively recent study, Knyazev [16] found that EEG oscillatory responses during personal moral decision-making differ significantly from those observed in both impersonal moral and non-moral conditions: the most pronounced differences were found in the delta and theta frequency bands, both of which are closely linked to emotional processing.

Also, the role of EEG hemispheric lateralization in the neural dynamics associated with moral judgment remains poorly understood. As regards neuroimaging studies, Cope et al. [17], for example, suggested that left–right asymmetry in the processing of not moral stimuli may be linked to the fact that morally relevant negative emotions are social emotions, which, unlike other negative emotions, are lateralized to the left hemisphere. Furthermore, they propose that while negative emotions may generally be lateralized to the right hemisphere, moral judgements may be lateralized to the left. Specifically, they propose that left-hemispheric involvement may be associated more with the judgment of potential moral violations than with the evaluation of the stimulus itself. On the contrary, to the best of our knowledge, there are no specific studies conducted by EEG on the lateralization during joint moral tasks.

Despite the lack of studies specifically examining the EEG dynamics of moral decision-making, numerous investigations have explored the involvement of various frequency bands in social and emotional processing, offering indirect insights into the oscillatory mechanisms potentially underlying moral cognition.

EEG studies have demonstrated that specific frequency bands, notably low-frequency bands, play a role in social processes, with oscillatory activity reflecting various cognitive, emotional, and motivational functions [18,19]. For instance, the delta band has been associated with emotional regulation and the processing of both positive and negative social interactions, suggesting a role in the attentional orientation towards emotionally salient stimuli [20]; furthermore, changes in delta activity have been linked to shifts in internal emotional states and their regulation, as well as to the facilitation of empathy and interpersonal synchrony, with increased delta activity observed during emotional tasks and in social contexts [21,22]. In contrast, the theta band has been linked to motivation, conflict monitoring, processing of salient information, and the regulation of behavior in social negotiation tasks [19,23,24], including the evaluation and integration of beliefs in joint decision-making contexts [25,26]. The literature about moral decision-making also reports findings concerning emotional engagement in terms of autonomic activity [27,28]. For instance, individuals with higher heart rate variability (HRV) appear to be more inclined to adhere to moral rules than those with lower HRV. This tendency can be attributed to increased sensitivity to others’ needs and greater attentiveness to others’ well-being [29]; similarly, individuals with higher baseline HRV have been shown to make wiser and less biased social judgments in situations involving moral content [30], as well as lower utilitarian tendencies when evaluating harmful actions, showing a preference for deontological positions [31].

Given the importance of neural and autonomic correlates in emotional regulation, motivation, and moral decision-making at the individual level, recent research has begun to explore how these processes occur in real-time social exchange. In particular, the hyperscanning paradigm [32] has been increasingly used in social neuroscience, allowing the non-invasive, simultaneous recording of neural or autonomic activity in two or more individuals engaged in a joint task, and offering valuable insights into shared cognitive and emotional processes.

Even though research on interpersonal synchronization during shared moral decision-making is still in an exploratory phase, numerous hyperscanning studies have investigated emotional and cognitive engagement in negotiation and cooperation tasks. For instance, a hyperscanning study by Angioletti et al. [2] explored the electrophysiological correlates of a negotiation task, suggesting that the process may involve independent cognitive regulation between the two individuals rather than complete neural synchrony. Acconito et al. [33] investigated negotiation through the integration of EEG and functional near-infrared spectroscopy (fNIRS), emphasizing the cognitive and neural correlates of the process, and identifying negotiation as a dynamic and interactive mechanism that supports joint decision-making. Furthermore, research conducted with fNIRS has indicated that collaborative decision-making is supported by neural activations and interbrain synchrony patterns that differ from those involved in individual decision-making. It has been observed that these processes are modulated by social context and individual differences [4,34]: the activation of the prefrontal cortex (DLPFC) and the middle frontopolar areas has been observed during collaborative decisions.

In addition, a growing body of research has indicated that autonomic synchronization between individuals can serve as an indicator of a high level of cooperative engagement and the perception of mutual influence during social interactions [35]. Findings suggest that joint decision-making processes are supported by autonomic alignment, which facilitates the collaborative development of moral actions. Conversely, divergence in autonomic patterns may indicate reduced engagement, diminished reciprocity, or greater relational asymmetry [36].

This theoretical framework suggests that shared moral decision-making processes are characterized by complex dynamics integrating cognitive and emotional components.

For example, anticipatory emotions (e.g., fear or anxiety) and anticipated emotions (related to expected consequences) have been shown to influence choices not only as heuristics but also by integrating with cognitive processing through bodily signals and physiological responses [37]. In addition, in social contexts, empathy, guilt, or indignation guide risk assessment and promote altruistic choices, highlighting the regulatory role of emotions [25,38]. These processes may also be influenced by the decision-maker’s preferred decision-making style, which may be more oriented towards either the emotional or cognitive dimension. Specifically, those who adopt a more cognitive decision-making style engage deliberative and counterfactual systems, whereas those adopting an affective decision-making style rely on intuitive evaluations, based on empathy [39]. This view is further supported by a recent study by Crivelli et al. [40], which, using a decision-making task similar to the one employed in the present study, found that individuals’ tendencies to rely more on emotional or cognitive information during decision-making are influenced by their personality traits. Specifically, individuals who preferred an emotional decision-making style demonstrated higher emotional stability and awareness, while others showed a preference for rational or intuitive styles. These differences were also confirmed by EEG and HRV correlates, demonstrating that a subjective preference for an emotional or cognitive decision-making style is reflected in distinct neural and physiological signals. For example, frontal theta activity was associated with a dependent style, whereas temporo-parietal theta activity correlated with a rational style. Also, higher HRV has been linked to better self-regulation, while lower HRV, which is associated with avoidant styles, indicates difficulties in emotional regulation.

Nevertheless, the existing literature exhibits notable gaps concerning the collaborative construction process of moral judgment, understood here as a cooperative, negotiation dynamic between individuals aimed at reaching a common course of action, both at neural and autonomic levels. Moreover, there is a limited exploration of interactive dynamics at both the EEG and autonomic levels in dyadic moral decision-making scenarios; in fact, to the best of our knowledge, only one study by Allegretta et al. [41] has explored specifically moral decision-making through an EEG hyperscanning task, considering the interplay of emotional and cognitive factors on effective cooperation.

The present study contributes to the growing field of hyperscanning studies on moral decision-making, which has so far predominantly focused on the individual level. It explores the neurophysiological synchronization mechanisms during a shared moral negotiation task, with particular attention to the associated cognitive and emotional dynamics. In order to achieve this purpose, each participant was first asked to choose one of four statements (two emphasizing emotional aspects and two emphasizing cognitive aspects) that best described their intended course of action, in order to classify them in relation to their initial decision-making orientation and distinguish between homologous (similar orientation) or heterologous (different orientation).

Then, pairs of participants were presented with a moral scenario of a health emergency, and were asked to reach a shared decision through a dialogical exchange process, while their EEG and autonomic correlates were recorded. Based on the existing literature on cognitive-emotional integration processes in decision-making tasks and affective regulation, it is hypothesized that, during the moral task, lower neural dissimilarity will be observed in low-frequency bands, particularly in frontal regions in the homologous dyads (i.e., dyads with similar emotional or cognitive orientation). This expectation is grounded on the premise that low-frequency bands within these regions play a pivotal role in negotiation, disagreement management, processing of salient information, and emotional and behavioral regulation in social contexts, which are fundamental in the establishment of a shared decision [19,25,26]. Furthermore, it is hypothesized to observe lower neural dissimilarity in the left hemisphere, because of its involvement in morally relevant negative emotions and the evaluation of potential moral violations, reflecting the sharing of more similar personal moral opinions [17].

Regarding autonomic dissimilarity, a significant effect is expected concerning HRV, which is considered a sensitive index of interaction quality and the degree of physiological coordination in collaborative contexts. Specifically, it is hypothesized that homologous dyads may exhibit lower HRV dissimilarity, as their shared decision-making styles may reduce the regulatory effort required to reach a shared agreement [42].

## 2. Materials and Methods

### 2.1. Sample

This study included a total of 30 university volunteer students (mean [M] age = 23.57 years, Standard Deviation [SD] of age = 1.95), organized into eight female dyads and six male dyads. Participants were recruited using a non-probabilistic convenience sampling strategy.

An a priori power analysis was performed using G*Power (version 3.1.9.7; ref. [43]) to estimate the required sample size for the planned repeated-measures ANOVA, assuming a large effect size (f = 0.40). The analysis indicated that a minimum of 12 units of analysis would be required to achieve a statistical power of 0.85 at an alpha level of 0.05. Consequently, the final sample size of 30 participants was deemed sufficient to ensure adequate power for detecting the expected effects. Therefore, the final sample size of 30 participants was considered appropriate for the planned analyses.

Exclusion criteria encompassed: having a history of neurological or psychiatric disorders, significant depressive symptoms, cognitive impairments, or memory deficits, or taking psychoactive drugs.

All participants signed the consent form before the experiment. The research was conducted in full accordance with the ethical standards set out in the 2013 Declaration of Helsinki and complied with the General Data Protection Regulation (GDPR, EU Regulation 2016/679). Ethical approval for the study was granted by the Ethics Committee of the Department of Psychology at the Catholic University of the Sacred Heart in Milan, Italy.

### 2.2. Experimental Procedure

Before the beginning of the experiment, each participant was formally instructed on the study procedures. To promote natural interaction and reduce potential distractions, participants were seated in fixed chairs close to each other and within sight of each other to allow for direct eye contact. Furthermore, to ensure effective communication, participants were asked to avoid whispering, to remain composed, and to respect their turn in the conversation, to prevent overlapping speech.

In addition, video recordings were utilized to collect any changes in non-verbal communication, such as facial expressions and body movements, throughout the session. The neural and autonomic activity of each dyad was recorded using a BIO-EEG hyperscanning setup, which enabled the simultaneous recording of brain and body activity. Before the experimental task, a 120 s resting baseline was collected.

#### Moral Task

Both members of the dyads were presented with a moral task (MT), inspired by the critical circumstances that occurred during the COVID-19 pandemic, in which a doctor, working in a hospital severely affected by the health emergency, is forced to decide which of two elderly patients, both in their eighties, should receive priority treatment.

The moral scenario was the following:

*“A physician at one of the hospitals facing the health emergency caused by the SARS-CoV-2 virus finds him or herself confronted with the difficult decision of who to prioritize for treatment between two elderly patients, both in their eighties. The first is a hypertensive patient who is widowed and receives care from his daughter, who is suffering from a chronic illness; the second is a patient with diabetes.”*

Participants were instructed to read the scenario and to assume the perspective of the doctor faced with the moral dilemma. Then, they were asked to answer the following question:

*“If you were the doctor, which of the two patients would you have given priority to?”*

By selecting the sentence that best describes their personal point of view among these four options:

1. *Priority is given to the first patient for the supporting role towards the sick daughter;*
2. *Priority is given to the first patient with greater severity of hypertension than diabetes;*
3. *Priority is given to the second patient due to their greater chance of survival;*
4. *Priority is given to the second patient due to the possibility of rapid improvement and discharge.*

The individual answers of the participants were recorded as “*individual* choice”.

The four sentences were designed to evaluate two separate aspects of moral reasoning: an emotional component (sentence 1 and 2), which highlighted interpersonal and empathetic considerations (such as family relationships and emotional strain), and a cognitive component (sentence 3 and 4), which centered on clinical outlook, treatment efficacy, and broader systemic implications. Participants were not informed about whether the statements were emotionally or cognitively oriented, to reduce potential priming and avoid biased responses.

**Figure 1:**
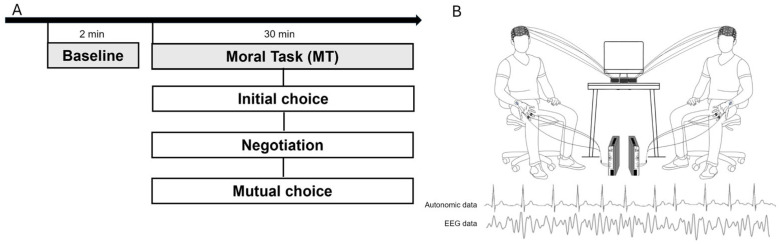
(**A**) The picture displays the timeline of the experimental procedure encompassing the EEG recording during the resting state baseline and the Moral Task (MT). (**B**) The picture displays the hyperscanning setup, where both the EEG and the autonomic data were collected simultaneously.

Following this, participants were requested to negotiate, within a maximum of three minutes, their initial choice to reach a mutual agreement on a unique sentence from the proposed options, which best describes their joint decision. The concluding sentences, which were the result of the negotiation process, were recorded as “*mutual* choice” (Figure 1).

For the analysis, a two-level variable “DyadOrientation” was created to distinguish between homologous and heterologous dyads, serving as a between-subjects factor in subsequent analyses. Homologous dyads are those in which both members initially chose sentences with the same orientation (emotive-emotive or cognitive-cognitive; N = 4), while heterologous dyads are those in which members initially chose sentences with different orientations (one emotive and the other cognitive; N = 11).

### 2.3. Data Collection and Processing

#### 2.3.1. EEG Data Acquisition and Processing

EEG data were collected during the 120 s baseline (BL) and the task (the MT). The NEUROSCAN 4.2 software was used in combination with a 16-channel DC amplifier (SYNAMPS system). Fifteen Ag/AgCl electrodes were placed according to the international 10/20 system [44] through an ElectroCap, with reference to the ears. The electrodes were placed in: Fp1, Fp2, F3, F4, Fz, Cz, C3, C4, T7, T8, Pz, P3, P4, O1, and O2. Furthermore, two EOG electrodes were applied to outer the canthus and below the left eye to detect eye movements. The inclusion of these electrodes enabled the identification and removal of EEG signals compromised by eye artifacts. The EEG signals were sampled at 1000 Hz and processed using a notch filter set at 50 Hz to limit electrical interferences. The impedance of the electrodes was maintained at a level below 5 kΩ. To pre-process the signal, the Brain Vision Analyzer 2.0 software (Brain Products GmbH, Gilching, Germany) was utilized. The experimental setup involved the application of an IIR bandpass filter (48 dB/octave) within the frequency range of 0.01 to 50 Hz. The continuous signal was then divided into two-second epochs. Independent Component Analysis (ICA) was performed to identify and remove components associated with ocular and muscular artifacts [45]. The ICA was applied conservatively, and only components clearly identified as artifacts (e.g., eye blinks, horizontal eye movements, muscle activity) were removed, following established guidelines. Typically, only a few components per participant were discarded, while all components reflecting neural activity were retained.

Following ICA, all epochs were visually inspected, and segments still contaminated by eye movements, muscle activity, or other disturbances were rejected (average 3% of epochs excluded). This indicates a high overall signal quality and supports the robustness of the preprocessing pipeline.

The power spectral density (PSD) was computed for both the BL and the task using a Fast Fourier Transform (FFT) with a Hamming window and a resolution of 0.5 Hz. For the negotiation task, only epochs corresponding to actual speech periods were included, ensuring that only artifact-free segments associated with verbal interaction were analyzed. The present study focused on five standard EEG frequency bands to conduct a PSD analysis. Delta (0.5–3.5 Hz), Theta (4–7.5 Hz), Alpha (8–12.5 Hz), Beta (13–30 Hz), and Gamma (30.5–50 Hz). The statistical analysis of the joint moral negotiation effect was quantified using a normalized index relative to the BL condition. The calculation of this ratio is as follows: [PSD_MT − PSD_BL]/PSD_BL. Furthermore, three Regions of Interest (ROI) were defined by aggregating data from the following electrode clusters: frontal region (Fp1, Fp2, F3, F4), temporo-central region (C3, C4, T7, T8), and parieto-occipital region (P3, P4, O1, O2). Hemispheric lateralization was also considered in this study.

#### 2.3.2. Autonomic Data

Electrocardiographic (ECG) signals were recorded using electrodes placed on the participants’ lower forearms, with the anode on the left side and the cathode on the right. Electrodermal activity (EDA) was measured with sensors placed on the thumb and little finger of the non-dominant hand, to ensure participant comfort and minimize interference with the hyperscanning equipment. ECG signals were digitized at a sampling rate of 1000 Hz and processed with a band-pass filter between 0.05 Hz and 35 Hz to remove background noise. The cardiac indices, including heart rate (HR) and heart rate variability (HRV), were then extracted following visual inspection and artifact removal to correct body movements or physiological interference. Heart rate variability (HRV) was quantified using the standard deviation of normal-to-normal intervals (SDNN), a time-domain measure reflecting total variability in cardiac activity. Furthermore, EDA signals were acquired at 1000 Hz, filtered with a 10 Hz low-pass filter to reduce muscle or eye-movement artifacts, and then converted to skin conductance level (SCL).

All signal acquisition and analysis were carried out using AcqKnowledge software (version 3.7.1, Biopac Systems Inc., Goleta, CA, USA). The normalization of all the autonomic metrics was performed, employing the same procedure for each participant within each pair as utilized in the EEG data analysis, using the formula [BIO_MT − BIO_BL]/BIO_BL to reduce the influence of pre-existing differences between dyads.

### 2.4. Data Analysis

To quantify the dissimilarity between cerebral and autonomic activation in each participant within a dyad, the Euclidean Distance Index (ED) was calculated for each measure. ED quantifies the absolute differences between members of a dyad in neurophysiological measures, and is commonly used in hyperscanning studies to assess interpersonal dissimilarity [2]. ED was calculated for each frequency band (delta, theta, alpha, beta and gamma), for each region of interest (ROI-F, ROI-TC, and ROI-PO), with electrodes grouped within ROIs and matched by hemisphere (lateralization). The same approach was applied to HRV, with ED computed between dyad members to quantify inter-individual autonomic dissimilarity. This provided an inverse index of neurophysiological similarity: higher values indicate greater dissimilarity and lower values indicate lower dissimilarity (i.e., greater alignment).

Five repeated measures ANOVA with a mixed design were conducted, one for each frequency band (delta, theta, alpha, beta, and gamma). In these analyses, Dyad Orientation (2: homologous, heterologous) was considered a between-subject factor, while region of interest (ROI; 3: frontal, temporo-central, parieto-occipital) and lateralization (2: left, right) were considered within-subject factors. The dependent variable in each model was the ED computed for EEG activity.

Regarding the autonomic correlates of the MT, three one-way ANOVAs were performed with Dyad Orientation (2: homologous, heterologous) as the between-subject factor and ED values as the dependent variables, computed for each autonomic measure (HR, HRV, and SCL).

In a preliminary statistical phase, the assumption of normality was assessed through skewness and kurtosis analyses, which confirmed that all data were normally distributed (*p* > 0.05). Significant interactions were investigated using pairwise comparisons to assess simple effects, and potential biases arising from multiple comparisons were addressed by using the Bonferroni correction. Therefore, probability values (*p*-value) were adjusted by the statistical software by multiplying the observed uncorrected *p*-value for each pairwise comparison by the number of comparisons made. Below-reported *p*-values for pairwise comparisons are adjusted values. Effect sizes were reported using eta squared (η^2^), and the significance level was set at α = 0.05.

## 3. Results

With the exception of one dyad, which exceeded the three-minute time limit, all pairs successfully reached a mutual agreement on the moral task. The average total speaking time per participant during the negotiation phase was 64.6 s (SD = 21.20). No meaningful differences were observed in the duration required to reach consensus (minutes: M = 2.29; SD = 0.59).

### 3.1. EEG Frequency Bands Results

Concerning the EEG results, a significant main effect in ROI was observed in the delta frequency band (F_[2, 24]_ = 4.080, *p* = 0.030, η^2^p = 0.254), for which higher mean values were found in the frontal ROI compared to the temporo-central and parieto-occipital (ROI-F M = 1.274, SD = 0.565; ROI-TC M = 0.699, SD = 0.514; ROI-PO M = 0.780, SD = 0.358). However, this effect did not survive multiple comparisons.

**Figure 2:**
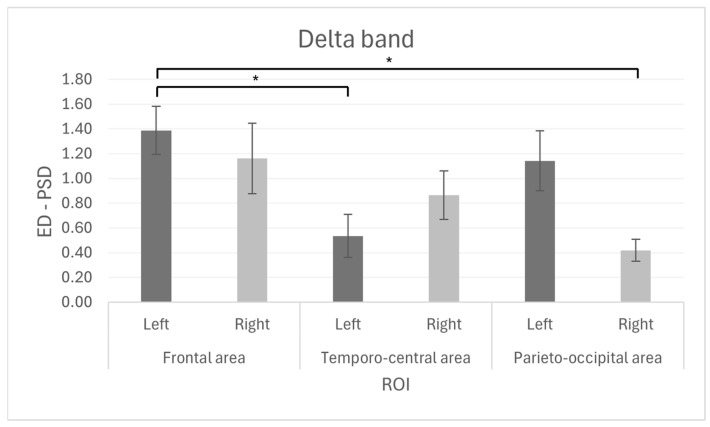
The bar chart shows the Euclidean Distance between the power spectral density (PSD) for the Delta band, for each ROI (Frontal area, Temporo-central area, Parieto-occipital area) and relative lateralization (Left, Right). Bars represent ±1 standard error, and stars (*) mark statistically significant effects.

Furthermore, a significant ROI × Lateralization interaction was observed (F_[2, 24]_ = 4.869, *p* = 0.017, η^2^p = 0.289). Pairwise comparisons indicated a greater dissimilarity in the left frontal region in comparison to the left temporo-central region (*p* = 0.007), as well as a greater dissimilarity in the left frontal region in comparison to the right parieto-occipital region (*p* = 0.003) (Figure 2). The 95% confidence intervals (CI) for each ROI and lateralization are reported in the text as follows: The 95% confidence intervals for each ROI × Lateralization condition were as follows: Frontal left [CI: 0.964–1.810], Frontal right [CI: 0.543–1.781], Temporo-central left CI:[0.156–0.914], Temporo-central right [CI: 0.437–1.290], Parieto-occipital left [CI: 0.614–1.668], Parieto-occipital right [CI: 0.224–0.614].

No significant effects were found for the other frequency bands (theta, alpha, beta, gamma).

### 3.2. Autonomic Indices Results

**Figure 3:**
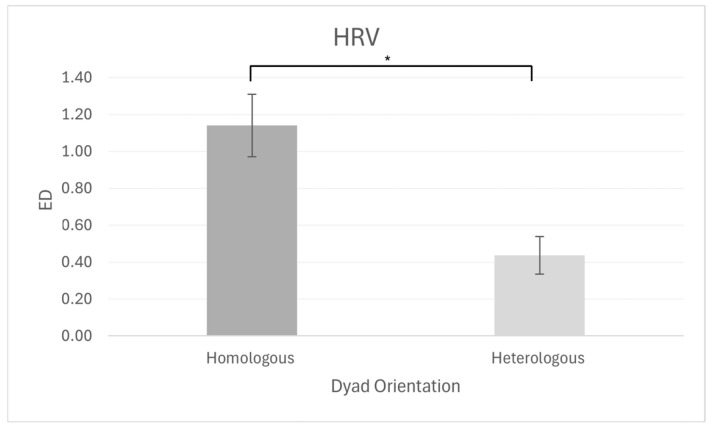
The bar chart displays the main effect of HRV, for which an increase in the dissimilarity index was detected for the homologous dyads compared with the heterologous ones. Bars represent ±1 standard error and stars (*) mark statistically significant differences.

As regards the autonomic indices, a significant main effect of dyad orientation was found for HRV (F_[1, 13]_ = 12.60, *p* = 0.004, η^2^p = 0.492), for which higher dissimilarity in HRV was observed in homologous dyads than in heterologous dyads (homologous [CI: 0.774–1.507]; heterologous: [CI: 0.216–0.658] (Figure 3).

No significant effects were found for HR or SCL.

## 4. Discussion

This multimeasure BIO-EEG hyperscanning study investigated the neurophysiological mechanisms underlying shared moral negotiation, with a focus on cognitive and emotional dynamics. Specifically, the study explored how potential differences in the initial decision-making orientations of the two members of each dyad (who were required to reach a joint moral decision) could influence neurophysiological synchronization during the moral negotiation process. Notably, the results did not confirm the initial hypotheses and, in some cases, exhibited contrasting patterns. For this reason, the following interpretations should be regarded as preliminary and exploratory.

First, EEG analysis revealed a significant effect in the delta band, specifically an interaction between cortical region (ROI) and lateralization, with greater dissimilarity observed in the left frontal ROI compared to the left temporo-central and right parieto-occipital regions. Contrary to our initial hypothesis, no significant effects were observed related to the dyads’ orientation.

These findings are consistent with the previous literature, which has linked delta band activity to affective-motivational processes during socially relevant decision-making tasks involving high emotional engagement, reflecting the integration of emotional states and moral evaluations [18,19,20,41]. In particular, activity observed through the left frontal sensors, in an area previously associated with decision-making and the processing of social emotions, may suggest the engagement of prefrontal mechanisms involved in the evaluation of emotional cost and sensitivity to potential moral violation [17]. In this context, the observed dissimilarity in this area may reflect the cognitive and emotional effort required to reach a shared agreement regarding which patient to save and, consequently, which one to sacrifice. This further supports the idea that such dissimilarity reflects a modulation of brain activity in response to the socio-emotional demands of the task, highlighting the role of delta band activity in morally salient interpersonal dynamics [20]. Also, the absence of an orientation effect may suggest that cooperation does not necessarily require neural (and cognitive) alignment but may instead involve distinct and parallel regulatory processes that are individually activated within a shared social context [46,47].

Conversely, the greater dissimilarity observed in the temporo-central and parieto-occipital regions may be indicative of the discrete functional roles of these areas. The temporo-central regions are linked to the processing of social signals, including biological motion and facial expressions. They are also associated with the management of semantic working memory, suggesting a more sophisticated processing of moral information. Conversely, the parieto-occipital region has been suggested to play a role in processes such as inferring others’ intentions and in attentional reorganization, thereby contributing to the understanding of others’ mental states and the evaluation of behavioral alternatives in the context of moral decision-making [48]. The expected effect in the theta band was not observed in the present study.

With regard to autonomic data, the results indicated a significant effect of dyad orientation on HRV dissimilarity. Contrary to expected outcomes, homologous dyads demonstrated greater dissimilarity in HRV patterns in comparison to heterologous dyads. This finding, which may appear to be counterintuitive, may potentially reflect the intrinsic meaning of this index. HRV represents an index of variability, so dissimilarity may indicate a reduced need for mutual adaptation in homologous dyads (where both members predominantly adopted an emotional or cognitive approach) and may therefore require less effort for regulation and coordination during negotiation. Sharing the same decision-making orientation could, in fact, reduce the necessity to align with one another, but increase the need to answer in a complementary way, allowing dyad members to regulate themselves more autonomously at the physiological level, thus exhibiting greater dissimilarity [2,36,49]. Conversely, in heterologous dyads (where one member was predominantly emotional and the other predominantly cognitive), the need to reach a shared decision from divergent positions may represent a heightened relational engagement and increased effort in negotiation dynamics and conflict co-regulation [36]. This potential explanation should be taken with caution given the limited sample size included in the study and the lack of correlation with behavioral metrics.

This study offers valuable insights into the hypothesis that moral decision-making in shared negotiation emerges from a complex interplay of cognitive and emotional processes, as well as regulation and co-regulation mechanisms. However, it is important to recognize that this study is not without limitations. Firstly, the limited sample size may restrict the generalizability of the findings and the capacity to comprehensively capture both the individual complexity and interpersonal interactions involved in the co-construction of moral judgment. Moreover, the distribution of participants was unbalanced, with fewer homologous dyads than heterologous ones. This suggests that the present work should be considered as exploratory, providing preliminary insights that require further confirmation in larger and more balanced samples.

Furthermore, given that the task was based on a realistic scenario that participants experienced, even if indirectly, it is possible that the results were influenced by the emotional involvement associated with the health emergency. Consequently, the hypothetical but highly specific nature of the presented context may restrict the applicability of the findings to real-life situations requiring decisions about others’ lives, such as clinical or medical settings. Another limitation is the use of a single task paradigm, which may limits the generalizability of the results. To test whether the observed mechanisms are consistent across different contexts, future studies should incorporate a broader set of moral dilemmas and decision-making scenarios beyond the single case of pandemic-related resource allocation.

Moreover, regarding EEG recordings, the current setup used a standard electrode arrangement with limited coverage, which provides only an approximate view of brain activity. Future research could benefit from denser electrode arrays or additional modeling approaches to improve spatial resolution and more accurately identify underlying neural sources. Furthermore, the utilization of the Euclidean distance index (ED) has facilitated the effective quantification of the overall amplitude dissimilarity between the signals of the dyad members. However, the results obtained through this method could be compared with those obtained through alternative metrics of inter-brain synchronization, such as spectral coherence or cross-correlation, in future studies. This would allow for the verification of the robustness and reliability of the results.

Finally, regarding HRV, we quantified it using SDNN, a time-domain measure reflecting overall heart rate variability. Given that HRV was extracted from fixed 3 min epochs, absolute values may not be directly comparable with studies using longer recordings. Future studies may benefit from employing longer recording windows and including complementary HRV metrics to provide a more comprehensive characterization of autonomic activity.

Furthermore, future research should consider the integration of subjective measures derived from validated psychometric questionnaires. The purpose of this integration would be to explore potential correlations between personality traits (e.g., openness to experience or emotional stability, as measured by instruments such as the Big Five Inventory [50], decision-making styles (intuitive versus analytical, as assessed by tools such as the General Decision-Making Style Questionnaire [51], and different dimensions of empathy, both cognitive and affective (e.g., as measured by the Index of Reactivity or the Balanced Emotional Empathy Scale [52,53]). In addition, future studies should aim to collect more detailed behavioral metrics related to negotiation, including the verbal interaction patterns, and decision dominance, to better explore links between neurophysiological measures and negotiation behavior. These variables could provide a valuable contribution to the understanding of individual differences in emotional regulation and co-regulation processes during the negotiation of complex moral dilemmas.

A further valuable direction for future studies would be to expand the sample to include greater heterogeneity in terms of age, cultural background, and professional experience, in order to better capture individual differences and interpersonal dynamics that influence moral negotiation more representatively. It would be of particular interest to administer the task to healthcare professionals, such as physicians and nurses, to observe decision-making dynamics in an even more ecological and practically relevant context. Indeed, these professionals are frequently confronted with complex moral dilemmas that have a direct impact on the lives and well-being of others. Incorporating professionals would also facilitate the evaluation of how training, clinical experience, and emotional habituation might influence moral negotiation capabilities and conflict management. This could be achieved through comparisons with non-professional populations, thereby providing valuable insights for the development of programs to enhance moral decision-making skills and empathetic awareness in high-responsibility professional environments.

## 5. Conclusions

In conclusion, the findings of this exploratory study suggest that shared moral negotiation emerges from the interplay of various factors, including cognitive and emotional processes, individual regulation and interpersonal co-regulation. While the initial hypotheses were not confirmed, observations regarding delta-band dissimilarity and heart rate variability (HRV) patterns suggest that collaboration may involve distinct and parallel processes adapted to the socio-emotional demands of the task. These results highlight the importance of considering both individual and interpersonal dynamics when trying to understand shared moral decision-making. However, given the exploratory nature of this study, its small sample size and the specificity of the adopted paradigm, these conclusions remain speculative and require further investigation using integrated methodologies that combine neurophysiological, autonomic, behavioral, and psychometric measures to delineate the mechanisms underlying moral negotiation in real-world contexts more effectively.

## Data Availability

The data presented in this study are available on request from the corresponding author due to ethical reasons for sensitive personal data protection (requests will be evaluated according to the GDPR-Reg. UE 2016/679 and its ethical guidelines).

## Abbreviations

The following abbreviations are used in this manuscript:

MT	Moral Task
ED	Euclidean Distance Index
